# Immune checkpoint gene *VSIR* predicts patient prognosis in acute myeloid leukemia and myelodysplastic syndromes

**DOI:** 10.1002/cam4.5409

**Published:** 2022-11-16

**Authors:** Kevin Yao, Emily Zhou, Evelien Schaafsma, Baoyi Zhang, Chao Cheng

**Affiliations:** ^1^ Department of Electrical and Computer Engineering Texas A&M University College Station Texas USA; ^2^ Department of Biosciences Rice University Houston Texas USA; ^3^ Department of Molecular and Systems Biology Dartmouth College Lebanon New Hampshire USA; ^4^ Department of Biomedical Data Science The Geisel School of Medicine at Dartmouth College Lebanon New Hampshire USA; ^5^ Department of Chemical and Biomolecular Engineering Rice University Houston Texas USA; ^6^ Department of Medicine Baylor College of Medicine Houston Texas USA; ^7^ Dan L Duncan Comprehensive Cancer Center, Baylor College of Medicine Houston Texas USA; ^8^ Institute for Clinical and Transcriptional Research, Baylor College of Medicine Houston Texas USA

**Keywords:** AML, MDS, prognosis, *VSIR*

## Abstract

**Background:**

Immune checkpoint proteins play critical functions during the immune response to cancer and have been targeted by immune checkpoint blockade therapy. V‐domain Ig suppressor of T cell activation (VSIR) is one of these immune checkpoint genes and has been investigated extensively in recent years due to its conflicting roles in cancer immunity. Specifically, in acute myeloid leukemia (AML), the prognostic value of *VSIR* is debated.

**Results:**

In both patient tumor samples and cancer cell lines we find that *VSIR* has the highest expression in AML out of all cancer types and, in AML, has the highest expression out of all other immune checkpoint genes. Survival analysis indicated that AML patients with higher *VSIR* expression have significantly shorter survival than those patients with lower expression, even within established AML subgroups (e.g., FAB subtypes). Importantly, *VSIR* expression is predictive of progression from myelodysplastic syndromes (MDS) patients into AML, suggesting its potential role during the very early stage of AML development and progression. In addition to AML, *VSIR* also demonstrates prognostic values in other cancer types, including multiple myeloma and mesothelioma.

**Conclusion:**

In summary, our analyses revealed the prognostic value of *VSIR* and its potential as a target for immunotherapy, especially in AML.

## Graphical abstract

The Kaplan‐Meier plot shows that patients with higher *VSIR* expression have a significantly worse prognosis. In addition, the forest plot showing that *VSIR* expression remains significant in predicting prognosis after adjusting for molecular and genetic variables like FAB score and cytogenetic risk.

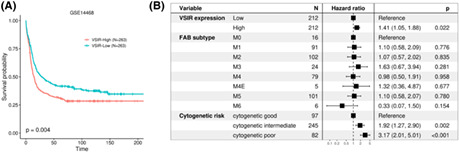

## 1 INTRODUCTION

Acute myeloid leukemia (AML) is a blood cancer of myeloid cells and is projected to have about 20,500 new cases and 11,540 deaths in 2022.^1^ AML is characterized by the presence of abnormal or poorly differentiated, proliferative, and clonal myeloid cells, leading to higher concentrations of myeloblasts in bone marrow, blood, and other tissues. These blasts consume body resources and prevent the genesis of normal healthy cells. AML often progresses quickly, and the 5‐year survival for AML patients is as low as about 30%.^1^ AML is more likely to develop in patients with underlying myelodysplastic syndromes (MDS), a heterogeneous class of diseases that result in ineffective hematopoiesis. In fact, about a third of patients with MDS will develop AML.^2^ The distinguishing criteria for MDS or AML diagnosis are the percentage of blasts in the bone marrow and peripheral blood, which has been arbitrarily set as 30% by the French‐American‐British (FAB) classification system^3^ and 20% by the World Health Organization (WHO).^4^

The main treatment for AML has traditionally been chemotherapy and targeted therapy drugs.^5^ In recent years, cancer treatment has been greatly advanced by the development of immunotherapy, including immune checkpoint inhibitors, adoptive cell therapies, and monoclonal antibodies.^6^ Such an advancement is largely attributed to the discovery of immune checkpoint targets, especially PD‐L1 and CTLA‐4. Therapy via blockade of these targets has found success for solid cancers such as melanoma^7^ and non‐small lung cancer.^8^ For AML, there have also been pilot studies on the use of PD‐L1 checkpoint blockade. Ravandi et al. found that the median event‐free survival of patients treated with nivolumab in combination with idarubicin and cytarabine was not reached in the study, but a considerable number of patients experienced immune‐related adverse events.^9^ In addition, Daver et al. found that the overall response rate of patients treated with nivolumab in combination with Azacitidine was 33%, with 11% of patients experiencing grade 3–4 immune‐related adverse effects.^10^ Such immunotherapies have yet to be widely recommended for AML. This may be due to adverse effects or because AML has a lower mutation burden and a more suppressed immune system,^11^, ^12^ which tends to decrease the efficacy of immune checkpoint blockade therapy.

In addition to PD‐L1 and CTLA‐4, other immune checkpoint targets like V‐domain Ig suppressor of T cell activation (*VSIR*, also called VISTA, C10orf54, B7‐H5, PD‐H1) have also been discovered and investigated.^13^ *VSIR* is an integral membrane protein with an extracellular immunoglobulin domain, a stalk, a transmembrane domain, and a cytoplasmic tail.^14^ Under physiological conditions, *VSIR* is highly expressed in hematopoietic lineages and tissues rich in infiltrating leukocytes and has lower expression in non‐hematopoietic tissue. In hematopoietic lineages, VSIR is expressed CD14^+^ monocytes, neutrophils, myeloid CD11c^+^ DCs, and CD4^+^ and CD8^+^ T cells. *VSIR* is not expressed on CD19^+^ B cells or CD56^Hi^ NK cells. In mice, *VSIR* is highly expressed in tumor‐infiltrating leukocytes. Unlike PD‐1/PD‐L1, *VSIR* expression is restricted to cells from hematopoietic lineages.^15^ *VSIR* not only serves as an inhibitor of T‐cell response, it also serves as a regulator for signaling and activation of innate immune cells. For example, in cancer, autoimmune, and inflammatory diseases, VSIR inhibits the production of inflammatory cytokines and chemokines in myeloid dendritic cells and macrophages.^15^ It is mainly expressed in hematopoietic cells in humans. *VSIR* is encoded by a gene located at 10q22.1, which is entirely confined inside an intron of the CDH23 gene.^16^ *VSIR* has attracted additional research interest due to its conflicting roles as both an inhibitory and a stimulatory immune checkpoint protein.^17^ In AML, *VSIR* has been found to be highly expressed.^18^ Knockout studies in mice found that *VSIR* induced immune evasion and caused the observed proliferation of AML cells.^18^ This supports the candidacy of *VSIR* as a novel checkpoint target in AML treatment. Wang et al. found a positive correlation between the expression of *VSIR* in peripheral myeloid cells and the expression of PD‐1 in T cells, even though there is no evidence that these genes are directly regulated.^19^ Since high PD‐1 expression has been implicated in a worse prognosis for AML patients,^20^ we expect that *VSIR* expression might also be associated with patient prognosis. However, previous studies have reported inconsistent results on the prognostic association of *VSIR* expression in AML.^19^, ^21^, ^22^ For instance, Wang et al.^19^ found that *VSIR* expression did not correlate with prognosis, but Zhang et al. and Chen et al.^21^, ^22^ found that higher *VSIR* expression is related to poorer prognosis. In addition, there have not been reports on the prognostic value of *VSIR* in MDS, the pre‐AML disease.

In this study, we investigated the prognostic value of *VSIR* gene expression in human AML and MDS. Using the TCGA (The Cancer Genome Atlas) and CCLE (Cancer Cell Line Encyclopedia) datasets, we first investigate the expression levels of *VSIR* in AML compared to other cancers and other immune checkpoint targets. We then establish the prognostic value of *VSIR* in AML based on survival analysis and show its potential for improving patient stratification in conjunction with established clinical factors, such as FAB subtype and cytogenetic risk. We also investigate the expression and prognostic value of *VSIR* in patients with myelodysplastic syndromes (MDS). Lastly, we perform a comprehensive analysis to examine the prognostic association of *VSIR* across seven different blood cancers and 33 other cancer types.

## 2 MATERIALS & METHODS

### 2.1 Datasets used in this study

The Cancer Genome Atlas (TCGA) datasets for the various cancer types studied in this study were downloaded from Firehose (https://gdac.broadinstitute.org/). Specifically, for LAML, the dataset contains RSEM‐normalized gene expression data for 20,501 genes and 173 patient samples. Genomic mutation and copy number variation (CNV) data were downloaded from Firehose.^23^ The genomic mutation data were downloaded in mutation annotation format (MAF) files and contain mutation information for 24,058 genes and 197 samples. Patient mutation burden is calculated as the sum of all non‐synonymous mutations. The CNV data were downloaded as a segmented copy number variation (sCNA) file and contain the CNV information for 23,311 genes and 380 patient samples. CNV burden is calculated as the fraction of genomic regions with abnormal chromosome copy numbers.^24^

Other than the transcriptomic and genomic data from TCGA, the study has investigated the following gene expression datasets. The cell line data for 33 cancers were downloaded from the Cancer Cell Line Encyclopedia (CCLE) database (https://portals.broadinstitute.org/). The de novo AML dataset contains 526 total samples and was downloaded from the Gene Expression Omnibus (GEO) under the accession ID GSE14468. The MDS dataset contains 159 MDS and 17 wild‐type samples and was downloaded from GEO under the accession ID GSE58831. The processed single‐cell RNA‐seq dataset was downloaded from GEO under accession ID GSE116256. It contained scRNA data for 38,410 cells from 40 bone marrow biopsies. Those biopsies were obtained from 16 patients with AML and five healthy donors. In addition, we have analyzed a collection of cancer gene expression datasets from the PREdiction of Clinical Outcomes from Genomic (PRECOG) (http://precog.stanford.edu) database, including a total of 166 datasets with matched gene expression profiles and patient survival information for seven different cancer types. More detailed information for these datasets can be found in Table S1.

### 2.2 Survival analysis

Overall survival (OS) event status and time were provided in the TCGA dataset. Using this data, we constructed univariate and multivariate Cox regression models using the “coxph” function with default parameters from the R library “survival.” The univariate Cox regression models were used to determine the association between overall survival and *VSIR* expression. The multivariate regression models were used to determine the association between OS and the following covariates: *VSIR* expression, FAB score, and cytogenetic risk. The results of the multivariate Cox regression models were visualized in forest plots, which was constructed using “forest_model” function from the R package “forestmodel.” Since *VSIR* expression is a continuous variable, we used the median *VSIR* expression to divide the samples into two equal‐sized groups (High and Low *VSIR* expression). The Kaplan–Meier method was used to plot the survival curves, and the log‐rank test was used to determine the difference between the two curves and calculate their *p*‐value. Survival analysis was performed using the R library “survival” with otherwise default parameters. The Kaplan Meier method was done using the function survfit from R library “survival” with default parameters, and the function “ggsurvplot” from the R library “ggplot” was used to plot the survival curves.

### 2.3 Comparing *VSIR* expression in subgroups

To test if *VSIR* expression is different between three or more subgroups, such as the eight FAB subtypes or three cytogenetic risk categories, we performed one‐way ANOVA using the R function “aov” with default parameters. To determine if VSIR expression is higher in one subgroup compared to the others, such as if VSIR expression is higher in NPM1 mut patients compared to NPM1 WT patients, we performed the one‐sided Wilcoxon test using the “wilcox.test” function in R.

### 2.4 scRNA‐seq analysis of *VSIR* expression in healthy cells and AML cells

Single‐cell RNA seq data of healthy cells were analyzed using data downloaded from the Bloodspot database, which provides mRNA expression profiles for a comprehensive list of hematopoietic cells.^25^ We selected the HemaExp v1 dataset and filtered the genes to select the *VSIR* gene. Bloodspot then generated a diagram for the expression of *VSIR* depending on the cell type.

For analysis of *VSIR* expression in AML patients, we utilized the GSE116256 dataset, which contains scRNA‐seq data on 38,410 cells from 40 bone marrow biopsies from 16 AML patients and five healthy patients. We chose to exclude the five healthy patients from this analysis, which left us with 30,712 cells. The cells were categorized as one of 14 cell types and determined to be cancerous or healthy. Of all the cell types, the only cancerous cells detected in this dataset were granulocyte monocyte progenitors (GMP), hematopoietic stem cells (HSC), progenitor cells (Prog), promonocytes (ProMono), classical dendritic cells (cDC), and monocytes (mono).

### 2.5 *VSIR* expression in other blood cancers

The PRECOG dataset was used to determine the expression of *VSIR* in other blood cancers. This dataset contains curated data from 166 datasets, including gene expression data and survival data for around 18,000 patients. Specifically, PRECOG contained gene expression data on the following hematopoietic cancers: two datasets for chronic lymphocytic leukemia (CLL), two datasets for multiple myeloma, one dataset for Burkitt lymphoma, seven datasets for AML, two datasets for B‐cell acute lymphoblastic leukemia (B‐ALL), three datasets for diffuse large B‐cell lymphoma (DLBCL), and one dataset for follicular lymphoma. To consolidate the survival analysis results from all datasets within a cancer type, after calculating the *p*‐values using the log‐rank test for each dataset individually, we used Fisher's method to calculate the meta *p*‐value for each cancer type.

## 3 RESULTS

### 3.1 *VSIR* is the immune checkpoint gene with the highest expression and association with prognosis in AML

**FIGURE 1:**
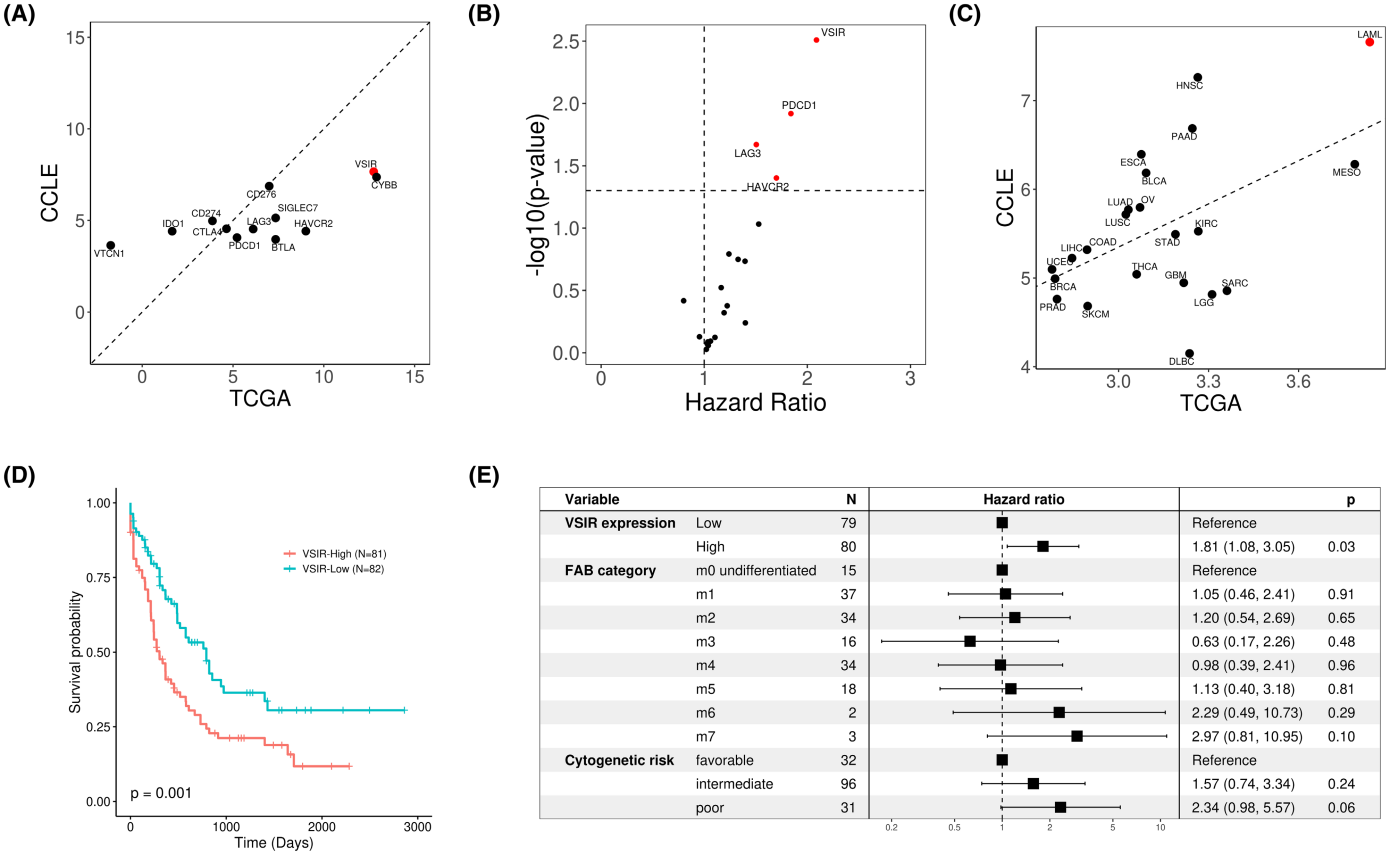
*VSIR* has highest expression and is prognostic in AML. (A) *VSIR* has the highest expression out of all cancer types in both the TCGA and CCLE datasets. (B) *VSIR* has the highest expression out of other known immune checkpoint genes in AML samples for both the TCGA and CCLE datasets. (C) Within TCGA AML samples, *VSIR* expression has the highest statistical significance and hazard ratio when predicting survival compared to other immune checkpoint genes. (D) Kaplan–Meier plot showing that TCGA AML patients with higher‐than‐median *VSIR* expression have significantly worser prognosis. (E) Forest plot validating the prognostic value of *VSIR* expression after considering the contributions of other molecular and genetic variables such as cytogenetic risk and FAB subtype in a multivariate Cox proportional hazards model.

To investigate the implication of immune checkpoint genes in leukemia, we compared their expression in AML patient samples from TCGA and leukemia cell lines from CCLE. The TCGA data reflect the mixed expression of genes in both tumor cells and non‐tumor cells in the tumor microenvironment, while CCLE data provide a proxy of gene expression in tumor cells only. As shown in Figure 1A, *VSIR* has the highest expression out of all the immuno‐checkpoint genes in both TCGA and CCLE datasets for AML. This indicates that *VSIR* expression is prominent in both cancer cells (CCLE) and the cancer microenvironment (TCGA), including immune cells that have infiltrated into the bone marrow. Furthermore, we examined the association of these immune checkpoint genes with the prognosis of patients with AML using the TCGA data. *VSIR* was determined to be the most prognostic immune checkpoint gene in AML (Figure 1B).

Previous studies have suggested that *VSIR* is highly expressed in hematopoietic tissues.^14^ To confirm this, we examined its expression pattern in different cancer types. Indeed, VISA shows the highest expression in AML out of all other cancer types in both TCGA and CCLE datasets (Figure 1C). We then differentiated TCGA AML samples into High and Low *VSIR* expression using the median as the threshold. The survival curves of the two groups are shown in Figure 1D. As shown, the *VSIR*‐high expression group has significantly shorter overall survival than the *VSIR*‐low expression group (*p* = 2 e‐3), with a median survival of 273 days compared with 471 days, respectively. Multivariate regression analysis indicated that *VSIR* expression remains significant after considering well‐known clinical factors, including the FAB category and cytogenetic risk group (Figure 1E), suggesting that it provides additional prognostic value to these established clinical features.

### 3.2 Validation of the prognostic value of *VSIR* in an independent dataset

**FIGURE 2:**
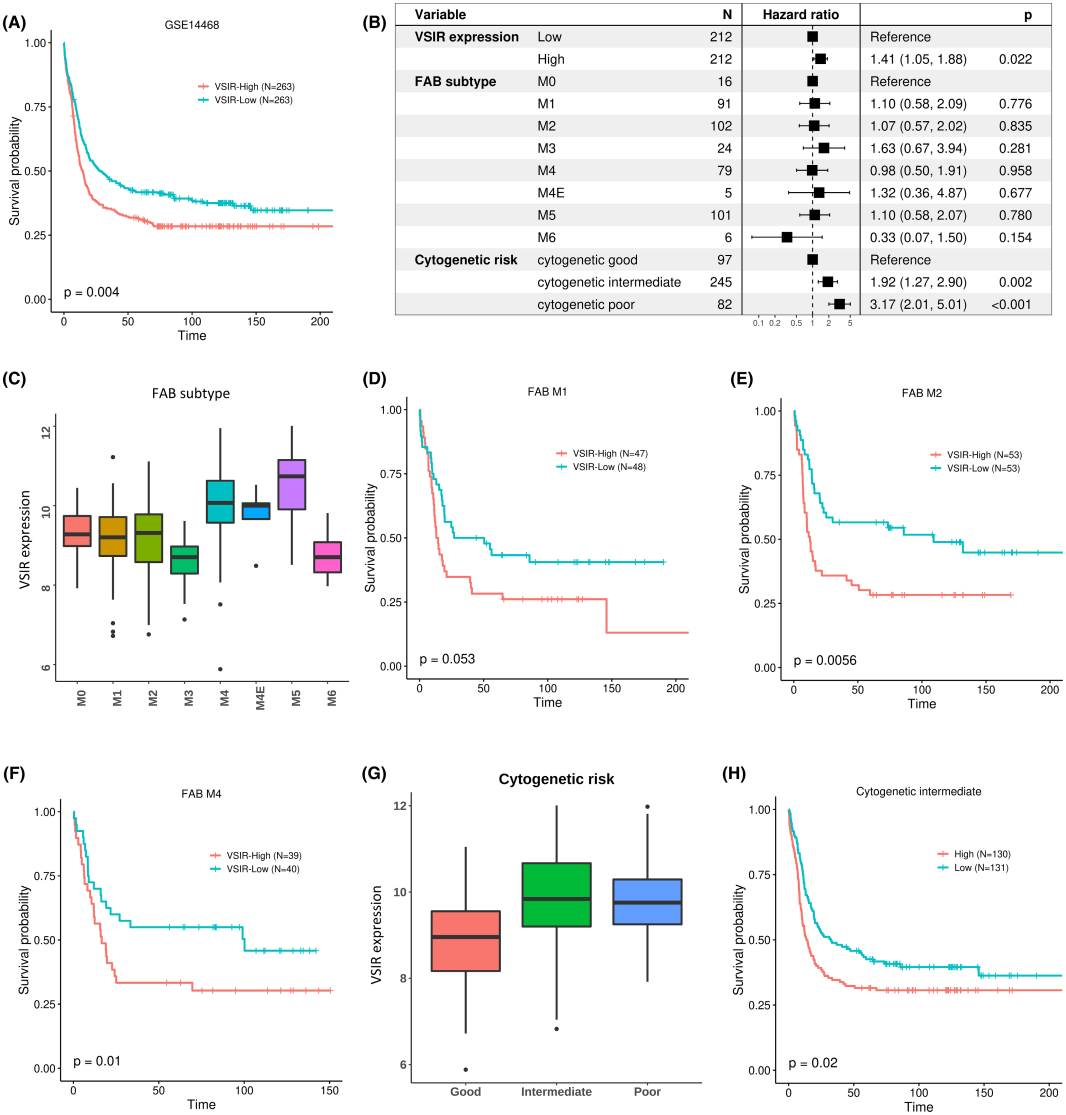
Validation of *VSIR* prognostic value in GSE14468. (A) Kaplan–Meier plot shows that patients with higher *VSIR* expression have a significantly worse prognosis. (B) Forest plot showing that *VSIR* expression remains significant in predicting prognosis after adjusting for molecular and genetic variables like FAB score and cytogenetic risk. (C) *VSIR* expression is different depending on the FAB subtype (ANOVA *p*‐value = 3 e‐39). (D–F) AML patients with the FAB M1, M2, and M4 subtypes, respectively, have significantly poorer prognoses if they have high *VSIR* expression. (G) *VSIR* expression is different depending on cytogenetic risk (ANOVA *p*‐value = 4 e‐17). (H) AML patients with an intermediate cytogenetic risk have a significantly poorer prognosis if they have high *VSIR* expression.

We further validate the prognostic association of *VSIR* in an independent AML dataset (GSE14468) with a larger number of samples (*n* = 526). Similar to previous results, after differentiating patients into two groups (High and Low *VSIR* expression) using the median as the threshold, patients with high *VSIR* expression exhibited significantly worse prognosis (*p* = 0.004) (Figure 2A), consistent with our previous observation. To account for possible confounding effects between *VSIR* and clinical variables, we performed a multivariate Cox regression analysis and found that high *VSIR* expression remained a significant prognostic indicator when clinical variables like FAB score and cytogenetic risk were considered (Figure 2B).

Biomarkers for cancer are often only applicable within specific molecular or clinical subsets of patients. For example, the Oncotype DX assay is only applicable to ER‐positive breast cancer patients.^26^ Considering this, we aimed to determine the prognostic value of *VSIR* within certain subsets of AML patients. One such classification system of AML is the French‐American‐British (FAB) system, which distinguishes roughly eight categories of AML based on cell type and maturity: M0 (myeloblastic without differentiation), M1 (myeloblastic with minimal maturation), M2 (myeloblastic with maturation), M3 (promyelocytic), M4 (myelomonocytic), M5 (monocytic), M6 (erythroid), and M7 (megakaryoblastic). *VSIR* expression varied considerably depending on the FAB subtype (ANOVA *p*‐value = 3 e‐39), suggesting that *VSIR* may play different roles within different subtypes. We thus systematically evaluated the prognostic value of *VSIR* within each subtype by utilizing Kaplan–Meier analysis on patients dichotomized by the median *VSIR* expression within that subtype. As demonstrated in Figure 2D–F, high *VSIR* expression in M1 (*p* = 0.05), M2 (*p* = 0.001), and M4 (*p* = 0.01) are associated with poor prognosis. In the other FAB subtypes, the prognostic association was not significant.

The next clinical subtype we considered was a cytogenetic risk (Figure 2G), which is a classification based on whether a patient's cytogenetics confer relatively favorable, intermediate, or poor prognosis. For example, patients with the t(8;21) translocation have a favorable prognosis, whereas the del(7q) abnormality confers poorer prognosis.^27^ Intermediate cytogenetic risk, which is exhibited in the majority of AML patients,^28^ appeared to have the highest *VSIR* expression, and higher *VSIR* expression is associated with significantly poorer prognosis (*p* = 0.01) (Figure 2H). Thus, we have demonstrated that high *VSIR* expression is associated with poor survival in both an external AML dataset and clinical variable categories commonly associated with AML.

### 3.3 *VSIR* is overexpressed in MDS patients and predicts overall and AML‐free survival

MDS has been previously referred to as a preleukemia disorder, and about 30% of MDS patients eventually develop AML.^29^ We, therefore, investigated the expression of *VSIR* in MDS samples. MDS patients exhibited significantly higher *VSIR* expression (*p* = 0.009) compared to their wild‐type or normal counterparts, which supports results from the previous work.^30^ Additionally, *VSIR* expression remains prognostic in predicting overall survival in both a univariate (*p* = 0.008) and multivariate cox proportional hazards model (*p* = 0.02) when considering established clinical variables like age, gender, and cytogenetic risk (Table 1). Because MDS has a high frequency of transition to AML, we next investigated if *VSIR* is able to predict AML‐free survival, where events are defined as a patient with MDS progressing to AML. We find that higher *VSIR* expression puts patients at higher risk for progression to AML in a univariate model (*p* = 0.05) and after adjusting for possible confounding effects of age, gender, and cytogenetic risk (*p* = 0.02, Table 1). These results support the theory that *VSIR* acts as an immune checkpoint inhibitor, which may allow proliferating blasts to escape immune detection and progress to AML.

**TABLE 1:** *VSIR* is prognostic for MDS patients

PMID	# Samples/Cancer types	Tissue type
25,079,552	173	AML Hematopoietic Tissue
22,460,905	33	Cell Line
26,193,342	7	Various
25,574,665	176	Bone Marrow
30,827,681	40	Bone Marrow
19,171,880	526	Bone Marrow

*Note*: *VSIR* expression significantly predicts overall survival and AML‐free survival for MDS patients. This is validated in both a univariate cox proportional hazards model and in a multivariate cox proportional hazards model considering clinical variables like age, gender, and cytogenetic risk.

### 3.4 Association of *VSIR* with the genomic landscape and biological pathways of AML patients

**FIGURE 3:**
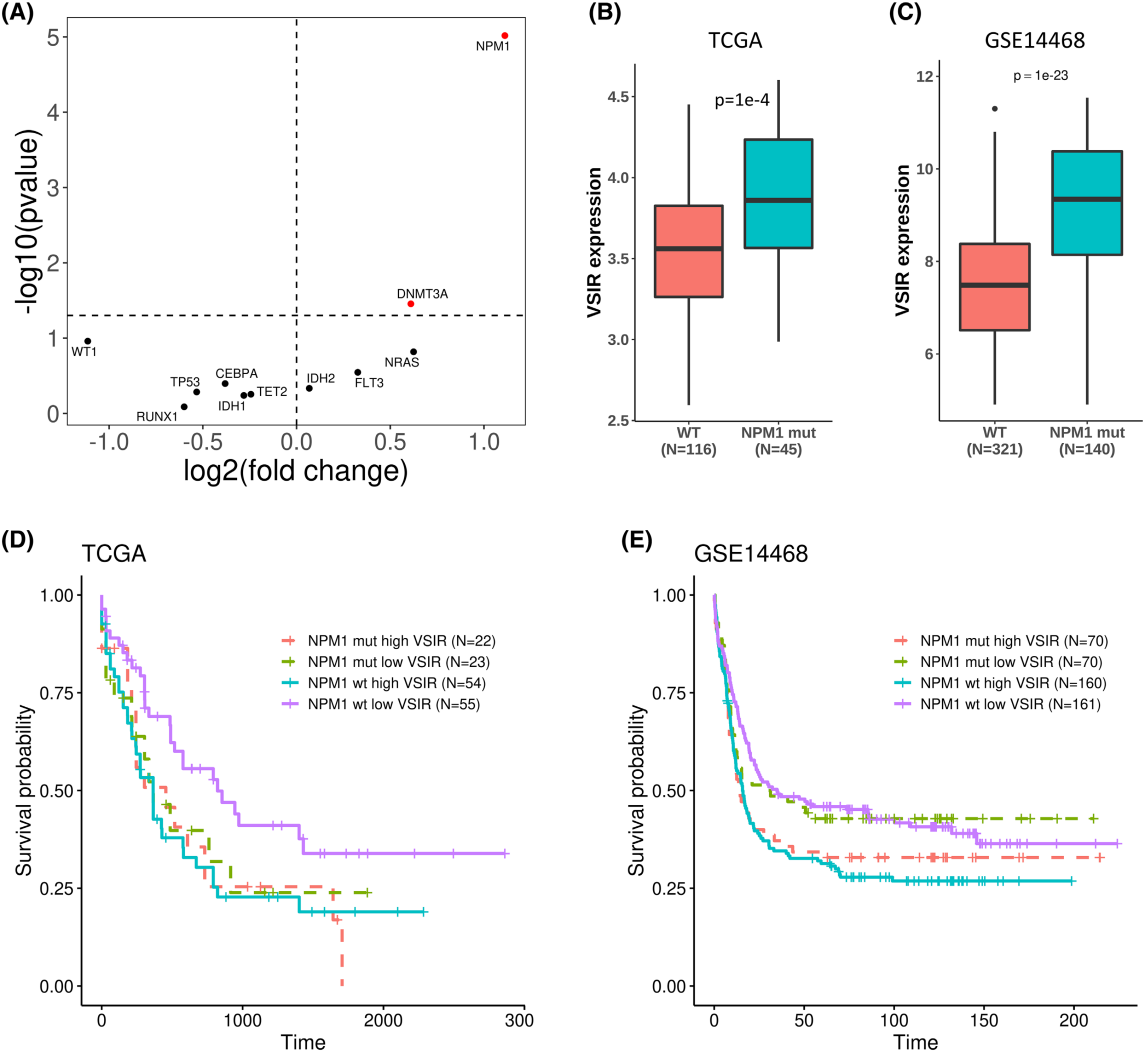
*VSIR* association with the genomic landscape. (A) Volcano plot showing that *NPM1* mutation is the only common genomic mutation in AML that has significantly different *VSIR* expressions between the group of patients with the mutation versus those without it. (B, C) Patients with the *NPM1* mutation have significantly higher *VSIR* expression versus those without the mutation in both the TCGA (Wilcoxon *p*‐value = 1 e‐4) and GSE14468 (Wilcoxon *p*‐value = 1 e‐23) datasets. (D, E) Kaplan–Meier plots showing that patients are significantly dichotomized by their *VSIR* expression if they are *NPM1* WT, but not if they have the *NPM1* mutation, in both the TCGA and GSE14468 datasets.

To further investigate whether the expression of *VSIR* is affected by upstream genomic aberrations, we determined the correlation between the patients' *VSIR* expression and their mutation burden or their CNV burden in the TCGA dataset. Using Spearman's correlation test, we found that *VSIR* expression is significantly associated with mutation burden (*p* = 0.006, *ρ* = −0.218) but not CNV burden (*p* = 0.5, *ρ* = −0.0545). To explore specific mutations that may be associated with *VSIR* expression, we chose to only study NRAS, DNMT3A, IDH1, TET2, *NPM1*, WT1, FLT3, IDH2, TP53, CEBPA, and RUNX1 mutations, which are all present in greater than 5% of AML samples in the TCGA dataset. We then tested whether *VSIR* expression is different between patients with the mutation and those without the mutation. The *p*‐values were calculated by the Wilcoxon test and were adjusted using the Bonferroni multiple testing correction to limit the false discovery rate. We plotted that against the *VSIR* fold change between mutated and wild‐type samples (Figure 3A). *NPM1* was the only mutation that remained significant, where patients with the mutation had higher *VSIR* expression (Figure 3B, *p* = 3 e‐5). This trend was also validated in the GSE14468 dataset (Figure 3C, *p* = 1 e‐23).

A previous study has reported that *NPM1* mutation was associated with a favorable prognosis,^31^, ^32^ but another study observed no significant prognostic association.^33^ Having shown that *VSIR* expression correlates with *NPM1* mutation status, we further investigated if the prognostic value of *VSIR* can be explained by the prognostic value of the *NPM1* mutation. We did not observe a significant survival difference between *NPM1* mutant and wild‐type samples in both TCGA and GSE14468 (Figure S1). In addition, we investigated the prognostic association of *VSIR* expression within the *NPM1* mutation and wild‐type subgroups. In the TCGA dataset, *VSIR* expression was negatively associated with patient prognosis (*p* = 0.01, HR = 1.837) in the *NPM1* wild‐type subgroup, but not in the mutant subgroup (*p* = 0.8, HR = 1.119) (Figure 3D). In the GSE14468 dataset, similar results were observed (Figure 3E). As shown, higher *VSIR* expression was a poor prognostic factor for patients in the *NPM1* wild‐type subgroup (*p* = 0.004, HR = 1.487), but high *VSIR* levels were not significantly associated with prognosis for *NPM1* mutant patients (*p* = 0.2, HR = 1.470). These results seem to indicate that *VSIR* expression may only be prognostic for patients without the *NPM1* mutation, but validation in a larger dataset is necessary to draw any definite conclusions.

Although *FLT3*‐ITD mutations were not significantly associated with *VSIR* expression, since they occur in roughly 20%–25% of AML cases,^34^ it is of importance to study the prognostic value of *VSIR* within populations with and without the *FLT3*‐ITD mutation. We found that *VSIR* expression significantly stratified patients without the mutation (*p* = 0.05, Figure S2A), but was unable to stratify patients with the *FLT3*‐ITD mutation (*p* = 0.4, Figure S2A), presumably due to the relatively small sample size (*N* = 63 for each group).

Additionally, we note that NPM1 mutant AML is a clinically heterogeneous subtype due to its frequent coexistence with other mutations. Specifically, the *FLT3*‐ITD mutation is a about twice as frequent in patients with *NPM1* mutations.^35^, ^36^ Thus, we next investigated the effect of *FLT3*‐ITD mutations on *VSIR* expression and prognostic value within *NPM1* mutant patients. We found that patients with the *FLT3*‐ITD mutation in tandem with the *NPM1* mutation had lower expression of *VSIR* (*p* = 0.05, Figure S2B). We then explored whether *VSIR* was prognostic within these subgroups and found that *VSIR* was not significantly prognostic within either (*NPM1* mutants with *FLT3*‐ITD mutations *p* = 0.53, *NPM1* mutants without *FLT3*‐ITD mutations *p* = 0.45, Figure S2C,D). The heterogeneity of *NPM1* mutants may be confounded by other factors outside *FLT3* mutations, such as stem cell signatures,^32^ so further research in a larger dataset is needed to confirm the prognostic value of *VSIR* within further subtypes of *NPM1* mutant samples.

### 3.5 The expression pattern of *VSIR* in different cell types

**FIGURE 4:**
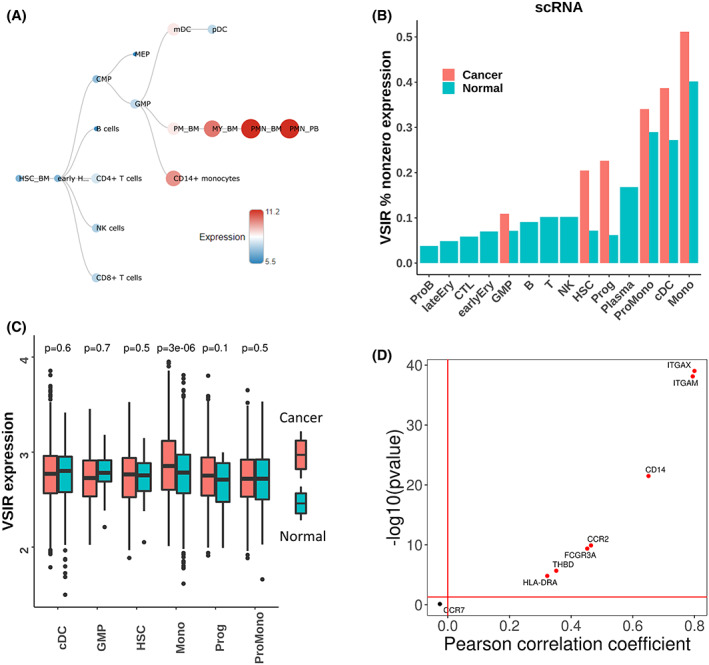
*VSIR* expression in single cells associated with leukemia. (A) *VSIR* expression in various hematopoietic cells of different cell lineages. Myeloid cells appear to have the highest expression of *VSIR*. (B) Single‐cell RNA sequencing data show that monocytes have the highest nonzero percent expression of *VSIR*. (C) Boxplots showing the distribution of *VSIR* expression for cDC, GMP, HSC, monocytes, progenitor cells, and pro‐monocyte cells based on whether the cell is healthy or cancerous. Malignant cells have consistently higher *VSIR* expression than normal cells. (D) *VSIR* expression correlation with monocyte marker genes such as ITGAM and CCR2. Any point above the horizontal red line has a significant nonzero Pearson correlation coefficient (*p*‐value <0.05).

To understand the potential mechanisms underlying the prognostic value of *VSIR* at the cellular level, we examined its expression pattern using scRNA‐seq data. First, we mapped the landscape of single‐cell *VSIR* expression for healthy patients using the HemaExplorer dataset based on their lineage in hematopoiesis (Figure 4A). As expected, cells of the myeloid lineage tend to have higher *VSIR* expression, especially monocytes and polymorphonuclear cells (PMN) from the bone marrow (BM) or peripheral blood (PB).

We next aimed to compare this landscape of normal *VSIR* expression to the *VSIR* expression levels of patients with AML by utilizing the GSE116256 dataset, which contains scRNA‐seq data on 30,712 cells from bone marrow biopsies from AML patients. We first aimed to identify the percentage of cells which express *VSIR* in these AML patients (Figure 4B). Cancerous cells generally have a higher nonzero expression of *VSIR*, and monocytes have the highest expression of *VSIR* for both normal and cancerous cells. We further explored the distribution of *VSIR* expression for normal vs cancerous cells with nonzero *VSIR* expression (Figure 4C) and found that monocytes are the only cell type where the *VSIR* expression in cancerous cells is significantly higher than that in normal cells. Based on this evidence, *VSIR* expression in monocytes is the most prominent and appears to be most affected when a person develops AML. Hypothesizing that *VSIR* may be co‐expressed on monocytes, we correlated the expression of *VSIR* with monocyte marker genes (Figure 4D) and found that every monocyte marker gene besides CCR7 is significantly correlated with *VSIR* expression. This further suggests that monocytes may be the major cell type that *VSIR* affects in AML patients.

### 3.6 *VSIR* expression is prognostic in other cancer types

**FIGURE 5:**
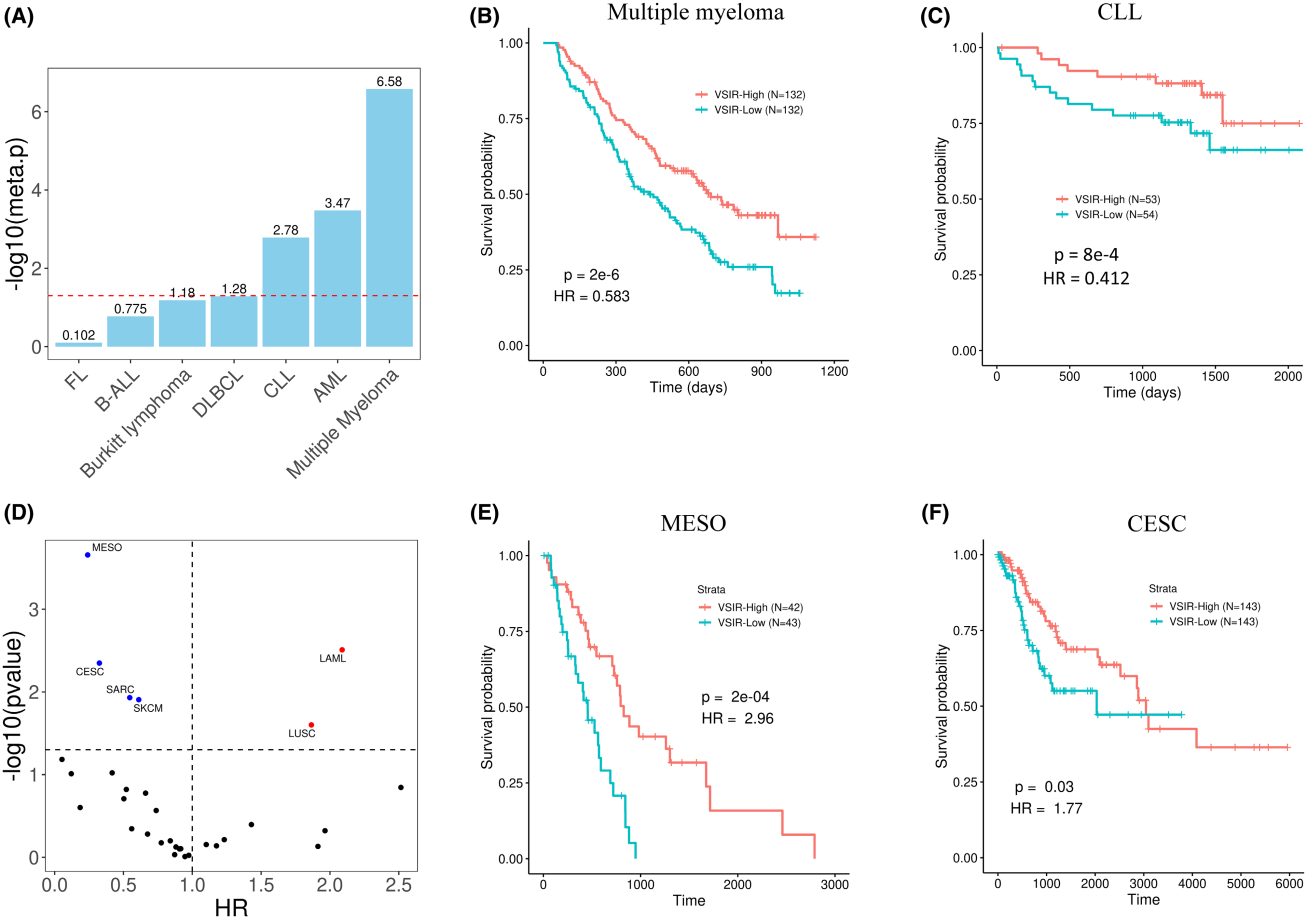
Prognostic value of *VSIR* in other blood cancer types. (A) Barplot showing the meta‐*p* values when predicting survival using *VSIR* in multiple myeloma, AML, CLL, and other blood cancers using the PRECOG dataset. (B, C) Kaplan–Meier plot showing that patients with higher *VSIR* expression have significantly better prognosis for both multiple myeloma and CLL, respectively. (D) Volcano plot showing that *VSIR* expression has high statistical significance when predicting survival in AML compared to other cancer types. (E, F) Kaplan–Meier plot showing that patients with higher *VSIR* expression have significantly better prognosis for both mesothelioma and cervical squamous cell carcinoma, respectively.

Finally, we explored the association of *VSIR* expression with prognosis in other blood cancers and cancer types. Initially, we examined other types of blood cancers. As shown in Figure 5A, *VSIR* expression is significantly prognostic in chronic lymphocytic leukemia (CLL), AML, and multiple myeloma. We further divided the samples into two groups by using the median *VSIR* expression and constructed a survival curve. We revealed that low *VSIR* expression is significantly associated with poor prognosis in multiple myeloma (*p* = 2 e‐6) (Figure 5B). Similarly, low *VSIR* expression is significantly associated with poor prognosis in CLL (*p* = 8 e‐4) (Figure 5C). After examining other types of blood cancers, we expanded our scope to include all the cancer types available on TCGA. After performing a univariate Cox regression model using overall survival and *VSIR* expression in each cancer type, it was revealed that *VSIR* expression has the highest statistical significance and hazard ratio in AML (Figure 5D). As shown in Figures 5E,F similarly to previous findings, low *VSIR* expression is associated with poor prognosis in mesothelioma (MESO) (*p* = 2 e‐4) and cervical squamous cell carcinoma (CESC) (*p* = 0.03). These results are consistent with the previous studies.^17^, ^37^, ^38^

## 4 DISCUSSION

In this study, we demonstrated that *VSIR* is prognostic and has the highest expression out of all other immune checkpoint genes in AML. We validated this claim in the TCGA and GSE14468 datasets and accounted for possible confounding clinical variables like FAB category and cytogenetic risk. Favorable cytogenetic risk predicted better survival, which aligns with current guidelines. *VSIR* score was also found to be prognostic within the FAB M1, M2, and M4 categories, as well as patients with intermediate cytogenetic risk. We also found that *VSIR* is significant in predicting both overall survival and AML‐free survival for MDS patients. Specifically, MDS patients with higher *VSIR* expression have a higher risk of regressing into AML. Next, we considered genomic driver events that correlate with *VSIR* expression and found that *NPM1* is the only common genomic mutation in AML that correlates significantly with *VSIR* expression. We then investigated the expression of *VSIR* in individual cells and found that monocytes have the highest expression of *VSIR* for both healthy cells and AML cells. Lastly, we determined that *VSIR* is also prognostic in other blood cancers like multiple myeloma and CLL and other cancers like mesothelioma and cervical squamous cell carcinoma.

Our study supports the theory that *VSIR* acts as an inhibitory immune checkpoint in AML.^17^ Higher *VSIR* expression suppresses immune responses at the cancer site and allows immune escape, leading to poorer prognosis. This should further support the ongoing efforts in determining the efficacy of *VSIR* blockade therapy in AML patients.^39^ Interestingly, we observe the same trends in MDS patients, suggesting that *VSIR* also suppresses immune response for MDS patients, which would cause poorer prognosis and a higher risk of progressing to AML. These results can have implications in MDS treatment. Currently, immunotherapy is not recommended for MDS treatment, but our results suggest that *VSIR* blockade therapy may yield benefits. Clinical trials of administering immunotherapy to MDS patients should be evaluated more carefully.

Currently, immune checkpoint inhibitors have modest efficacy in AML and MDS. The ORR rate of *PD1* inhibitor combined with azacitidine in RR‐AML is about 30%, and the CR rate is about 20%.^40^ Recently, a few clinical trials have been performed that evaluated the efficiency of anti‐*VSIR*‐based immunotherapy in multiple cancer types as monotherapy or combination therapies. Several *VSIR* inhibitors have undergone phase I and II clinical trials in solid tumors.^41^, ^42^, ^43^ The *VSIR* inhibitors include CA‐170 (NCT02812875), CI‐8993 (NCT04475523), and W0180 (NCT04564417). CA‐170, an anti‐*VSIR* and anti‐*PD‐L1* inhibitor, has shown very promising results. For example, in a phase II study for nonsquamous non‐small cell lung cancer, it was demonstrated that CA‐170 had a clinical benefit rate of 75% and patients had 19.5 weeks of progression‐free survival.^41^ However, none of these trials were tested for treating AML or MDS. Our analysis in this study indicates that *VSIR* has the highest expression levels in AML compared to other cancer types; and in AML *VSIR* has the highest expression than the other immune checkpoint genes. These results suggest that *VSIR* might be a good target for delivering effective immune checkpoint blockade therapy.

Importantly, for all cell types, *VSIR* expression increases when the cell develops AML, with monocytes having the highest *VSIR* expression with or without cancer. *VSIR* may directly bind to T cell receptors as a ligand, suppressing its activation.^44^, ^45^ Another possible pathway is that *VSIR* binds to and causes conformational changes in galectin‐9 that serve to enhance its effect.^46^ Galectin‐9 has been reported to limit the activity of natural killer cells and induce apoptosis of T cells, thus limiting their ability to respond against cancer.^47^, ^48^ Our results suggest that the mechanism behind why higher *VSIR* expression leads to poorer prognosis may be that cancerous cells produce more *VSIR* protein, which through binding to T cells and galectin‐9, inhibits immune response. Monocytes appear to be the biggest player in the expression of *VSIR*, which may be understood since monocytes were the only cells (out of naive T cells, CD4 central memory T cells, CD4 effector memory T cells, CD8 EM T cells, NK cells, B cells, and basophils) that experienced significant transcriptional signature changes when *VSIR* was bound by anti‐*VSIR* antibodies.^49^ Functionally, the transcriptional changes serve to enhance monocyte activity, such as increased secretion of IFNγ in a mixed lymphocyte reaction.

Lastly, we note that higher *VSIR* expression leads to a better prognosis in multiple myeloma, CLL, MESO, and CESC. This is contrasted by the observation that higher *VSIR* expression leads to poorer prognosis in AML. The survival advantage of higher *VSIR* expression in multiple myeloma, MESO, and CESC agrees with the previous work,^17^, ^37^, ^38^ but the prognostic value of *VSIR* in CLL has not been studied, to our knowledge. Both CLL and multiple myeloma are lymphoid cancers, which suggests that *VSIR* may play opposing roles depending on the hematopoietic lineage of cancer. Overall, this supports the consensus that *VSIR* plays a multifaceted role in cancer immunity across different cancers and may have co‐inhibitory or stimulatory roles in the immune response.^17^

Our study is currently limited by the lack of large datasets with *VSIR* expression data for AML patients. Our study may also be further improved through the incorporation of *VSIR* proteomics data, since gene expression may have a low correlation with protein expression.^50^, ^51^ A direct correlation between *VSIR* protein expression and poorer prognosis may further support the idea that *VSIR* should be targeted in checkpoint blockade therapy.

In conclusion, we have reported that *VSIR* is a poor prognostic factor for overall survival in AML and MDS. These associations hold after accounting for possible confounding clinical variables. *VSIR* is also prognostic within specific subgroups of patients. Importantly, MDS patients with higher *VSIR* expression are also more likely to develop AML. Not only do these results further support efforts in administering *VSIR* blockade therapy for AML patients, we also suggest that *VSIR* blockade therapy may be a promising treatment for MDS patients, which is a relatively unexplored prospect.

## AUTHOR CONTRIBUTIONS

**Kevin Yao:** Data curation (equal); formal analysis (equal); methodology (equal); writing – original draft (equal). **Emily Zhou:** Formal analysis (equal); visualization (equal); writing – review and editing (equal). **Evelien Schaafsma:** Writing – review and editing (supporting). **Baoyi Zhang:** Writing – review and editing (supporting). **Chao Cheng:** Conceptualization (lead); data curation (lead); formal analysis (supporting); funding acquisition (lead); methodology (lead); project administration (lead); supervision (lead); writing – review and editing (lead).

## FUNDING INFORMATION

This work is supported by the Cancer Prevention Research Institute of Texas (CPRIT) (RR180061 to CC). CC is a CPRIT Scholar in Cancer Research.

## CONFLICT OF INTEREST

The authors have declared that no conflict of interest exists.

## Supporting information


Figure S1

Figure S2

Table S1

## Data Availability

All data generated or analyzed during this study are included in this published article and its supplementary information files.
