# Supplementary material for: Immune checkpoint gene VSIR predicts patient prognosis in acute myeloid leukemia and myelodysplastic syndromes
Source: Cancer Med. 2022 Nov 16;12(5):5590–602. doi: 10.1002/cam4.5409 (PMC10028170; doi:10.1002/cam4.5409)
Supplement: Supplementary file 1 — Figure S1 [file CAM4-12-5590-s003.pdf]

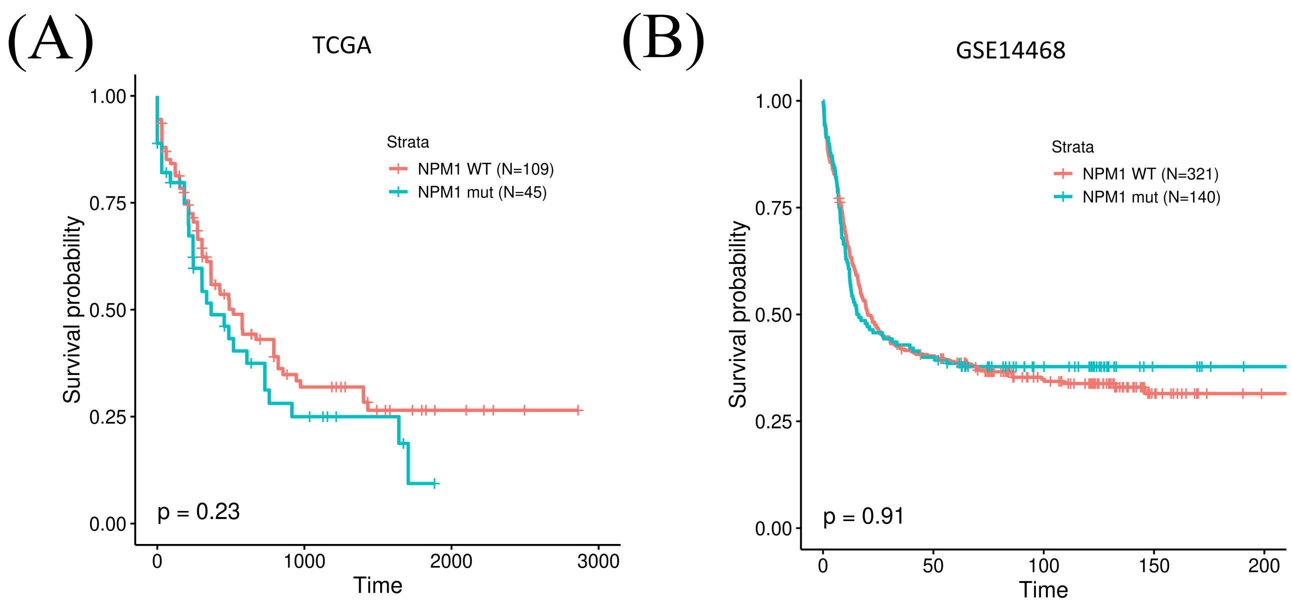


**Suppl. Fig. S1. NPM1 mutation is not prognostic**. Patients dichotomized by their NPM1 mutation status did not exhibit significantly different survival in the TCGA (A) or GSE14468 (B) datasets.
