# Supplementary material for: Immune checkpoint gene VSIR predicts patient prognosis in acute myeloid leukemia and myelodysplastic syndromes
Source: Cancer Med. 2022 Nov 16;12(5):5590–602. doi: 10.1002/cam4.5409 (PMC10028170; doi:10.1002/cam4.5409)
Supplement: Supplementary file 2 — Figure S2 [file CAM4-12-5590-s001.pdf]

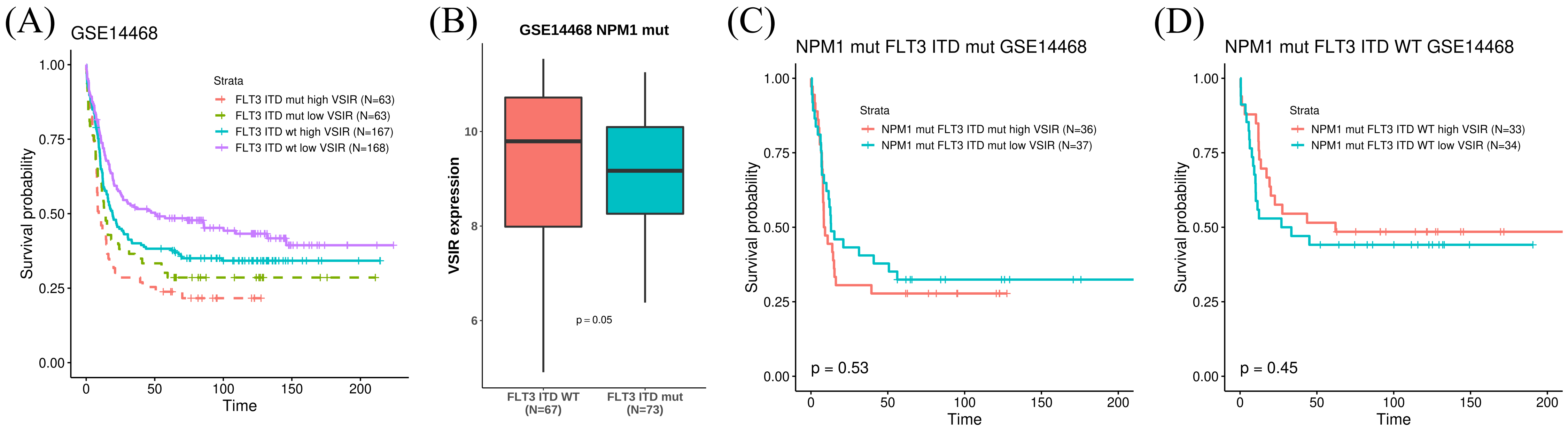

Suppl. Fig. S2. VSIR prognostic value in FLT3-ITD subgroups. (A) Patients are significantly stratified by their VSIR expression if they are FLT3-ITD WT, but not if they are FLT3-ITD mutant. (B) Given that a patient has the NPM1 mutation, those with the FLT3-ITD mutation have lower VSIR expression. (C-D) For patients with the NPM1 mutation, VSIR expression does not significantly stratify patients within the FLT3-ITD mutant or FLT3-ITD WT subgroups.
